# It Is Not Just Folklore: The Aqueous Extract of Mung Bean Coat Is Protective against Sepsis

**DOI:** 10.1155/2012/498467

**Published:** 2012-10-24

**Authors:** Shu Zhu, Wei Li, Jianhua Li, Arvin Jundoria, Andrew E. Sama, Haichao Wang

**Affiliations:** ^1^Laboratory of Emergency Medicine, The Feinstein Institute for Medical Research, 350 Community Drive, Manhasset, NY 11030, USA; ^2^Department of Emergency Medicine, North Shore University Hospital, The Hofstra North Shore-LIJ School of Medicine at the Hofstra University, Manhasset, NY 11030, USA

## Abstract

Mung bean (*Vigna Radiata*) has been traditionally used in China both as nutritional food and herbal medicine against a number of inflammatory conditions since the 1050s. A nucleosomal protein, HMGB1, has recently been established as a late mediator of lethal systemic inflammation with a relatively wider therapeutic window for pharmacological interventions. Here we explored the HMGB1-inhibiting capacity and therapeutic potential of mung bean coat (MBC) extract *in vitro* and *in vivo*. We found that MBC extract dose-dependently attenuated LPS-induced release of HMGB1 and several chemokines in macrophage cultures. Oral administration of MBC extract significantly increased animal survival rates from 29.4% (in saline group, *N* = 17 mice) to 70% (in experimental MBC extract group, *N* = 17 mice, *P* < 0.05). *In vitro*, MBC extract stimulated HMGB1 protein aggregation and facilitated both the formation of microtubule-associatedprotein-1-light-chain-3-(LC3-)containing cytoplasmic vesicles, and the production of LC3-II in macrophage cultures. Consequently, MBC extract treatment led to reduction of cellular HMGB1 levels in macrophage cultures, which was impaired by coaddition of two autophagy inhibitors (bafilomycin A1 and 3-methyladenine). *Conclusion*. MBC extract is protective against lethal sepsis possibly by stimulating autophagic HMGB1 degradation.

## 1. Introduction

Sepsis is an overwhelming systemic inflammatory response to severe infections, and can lead to shock, multiple organ failure, and death if not treated promptly. Despite recent advances in therapy, it remains the primary cause of mortality in medical intensive care units. Sepsis afflicts approximately 750,000 Americans each year, and costs the US healthcare system nearly $17 billion annually [1]. Current treatments are predominantly supportive and often ineffective. For instance, the only FDA-approved therapy for patients with severe sepsis was activated protein C (APC) [2], which was marginally effective and consequently removed from the market in 2011. Thus, the development of effective therapeutic interventions represents significant and yet unmet medical needs in the world. 

 The pathogenesis of sepsis is rather complex, but is partly mediated by endotoxin, which stimulates macrophages/monocytes to sequentially release early (e.g., TNF, IL-1, IFN-*γ*) and late (e.g., HMGB1) proinflammatory mediators [3–6]. In animal models of endotoxemia or sepsis, circulating HMGB1 increases to plateau levels between 24 and 36 h [5, 7], distinguishing itself from TNF and other early cytokines [8]. Furthermore, HMGB1-neutralizing antibodies confer protection against lethal endotoxemia [5] and sepsis [7, 9] even when given 24 h after the onset of sepsis, suggesting HMGB1 as a critically important late mediator of lethal sepsis [10]. Thus, therapeutic agents capable of inhibiting HMGB1 release [11–17] may hold potential for the treatment of lethal systemic inflammatory diseases. 

Autophagy (“self-eating”) is an evolutionarily conserved recycling process that various cells can employ to degrade endogenous cytoplasmic macromolecules to maintain cellular homeostasis [18]. It begins with the formation of microtubule-associated protein-1-light-chain-3-(LC3-) containing double-membraned cytoplasmic vesicles called autophagosomes. Subsequently, autophagosomes fuse with lysosomes to form degradative autophagolysosomes, where the engulfed contents are degraded by acidic lysosomal hydrolases [19]. Autophagy can be stimulated by starvation [20], pathogen-associated molecular patterns (PAMPs, such as endotoxin) [21], or cytokines (such as IFN-*γ*) [20, 22]. Recently, we discovered that green tea catechins (e.g., EGCG) suppressed endotoxin-induced HMGB1 release by stimulating its aggregation and autophagic degradation [23, 24]. It is presently unknown whether other medicinal herbs inhibit HMGB1 release and confer protection through similar mechanisms. 

 Mung bean (*Vigna radiata*) is widely consumed as a nutritional food in the forms of cooked whole beans, flour, or sprouts [25]. In China, it has also been used as a medicinal herb for dissipating fever and detoxicating the body since the 1050's. Interestingly, in line with the ancient description, recent studies have manifested that mung bean extract [26] and/or components (e.g., vitexin and isovitexin) [27] can alleviate pathogenic heat and oxidative stresses. In this study, we demonstrated that mung bean coat (MBC) extract remarkably inhibited endotoxin-induced release of HMGB1 and several cytokines/chemokines in macrophage cultures, and rescued mice from lethal sepsis. These effects may be attributable to MBC's capacity in stimulating HMGB1 aggregation and possibly autophagic degradation. 

## 2. Materials and Methods

### 2.1. Preparation of Aqueous Mung Bean Extract

Mung beans (MB) were purchased from a local market in New York. To prepare the whole bean extract, 10 g MB was boiled in 200 mL water for 20 min, and the water-soluble fraction was cleared and sterilized sequentially by centrifugation (3,100 g, 30 min, 4°C) and filtration (through a 0.22-*μ*m filter, Millipore Corporation, MA, Cat no. SLGP033RS). To obtain MB coat (MBC) extract, 30 g MB was soaked in water at room temperature for 24 h, and the MB coats were dissected. Subsequently, the MBC and the remaining flesh were separately boiled in 100 mL water for 10 min to obtain MB coat or flesh extract, respectively. The water-soluble fraction of MB coat or flesh extract was collected after sequential centrifugation and filtration as described above. From 30 g MB, approximately 0.3 g yellow substance was recovered from the MB coat extract after lyophilization. As chemical standards, vitexin (Sigma-Aldrich, MO, Cat# 49513) and isovitexin (Extrasynthese, France, Cat# 38953-85-4) were obtained from commercial sources. Bafilomycin A1 (Cat# B1793) was purchased from Sigma-Aldrich (St. Louis, MO). HMGB1-specific polyclonal antibodies were generated in rabbits as previously described [5]. LC-3 mouse monoclonal antibody was obtained from Santa Cruz Biotechnology. Anti-*β*-actin antibody was purchased from Sigma-Aldrich. 

### 2.2. Preparation of Recombinant HMGB1

The cDNA encoding for rat HMGB1 was cloned onto a pCAL-n vector, and the recombinant CBP-HMGB1 (rHMGB1) was expressed in *E. coli* BL21 (DE3) pLysS cells as previously described [5]. Contaminating endotoxin was removed from the HMGB1 preparation by Triton X-114 extractions as previously described [16]. To determine whether MBC or its major components (e.g., vitexin, or isovitexin) induced HMGB1 aggregation, rHMGB1 (30 mg/L) was incubated with MBC (20 mg/L), vitexin (40 mg/L), or isovitexin (40 mg/L) in 1x PBS (pH 7.4, 37°C) in the absence or presence of DTT (60 mM), and subsequently assayed for protein aggregation by SDS-PAGE followed by Coomassie blue staining, or Western blotting analysis.

### 2.3. Cell Culture

Murine macrophage-like RAW 264.7 cells and human monocyte U-937 cells were obtained from the American Type Culture Collection (ATCC, Rockville, MD). Human U-937 monocytes were differentiated into macrophages by incubation with 20 nM phorbol 12-myristate 13-acetate (PMA, Sigma-Aldrich, MO, Cat# P8039) for 3 days. GFP-LC3-transfected RAW 264.7 cells were established as previously described [24], and maintained in RPMI 1640/10% FBS/2 mM glutamine supplemented with puromycin (2 *μ*g/mL, Sigma, P9620, St. Louis, MO) to retain clonal homogeneity. Primary peritoneal macrophages were isolated from Balb/C mice (male, 7-8 weeks, 20–25 grams) at 3 days after intraperitoneal injection of 2 mL thioglycolate broth (4%) as previously described [28–30]. 

### 2.4. LPS and HMGB1 Stimulation

Adherent murine or human macrophages were gently washed with, and cultured in, OPTI-MEM I medium for 2 h before stimulation with endotoxin (lipopolysaccharide, LPS, *E. coli* 0111:B4, Sigma-Aldrich) or highly purified HMGB1 in the absence or presence of MBC extract, vitexin, or isovitexin at indicated concentrations. At 16 h after LPS or HMGB1 stimulation, intra and extracellular levels of HMGB1 were determined by Western blotting. In addition, the levels of other 42 cytokines in the conditioned cell culture medium were determined by Cytokine Antibody Array (RayBiotech Inc., Norcross, GA, Cat# AAH-CYT-3) as previously described [24]. 

### 2.5. Visualization of LC3-Containing Cytoplasmic Vesicles

The basic principle of autophagy assays was to measure the transfer of a soluble, membrane-impermeable LC3 protein from cytosol to autophagic vesicles (autophagosomes) [31]. To visualize LC3-containing cytoplasmic vesicles, GFP-LC3-transfected RAW 264.7 cells were stimulated with LPS in the absence or presence of MBC extract for 16 h and examined for the formation of GFP-LC3 punctate structures under a fluorescence microscope as previously described [24]. 

### 2.6. Western Blotting Analysis

The ratio between the 18-kD cytosolic LC3-I and 16-kD lipidated autophagosome-bound LC3-II was determined by Western blotting analysis as previously described [24]. The autophagic flux was measured by evaluating the effects of MBC extract on LC3-II turnover in the presence of an autophagy inhibitor, bafilomycin A1 at saturate concentrations. Briefly, macrophage cultures were stimulated with MBC extract (15 mg/L) and rHMGB1 protein (2 mg/L) for 4 h, and bafilomycin A1 (Sigma-Aldrich, MO, Cat# B1793) was added at a saturate concentration (100 nM) as previously described [24]. At 4 h post bafilomycin A1 addition, cells were harvested and assayed for LC3-II concentrations by Western blotting analysis with reference to a house-keeping protein, *β*-actin. To assess the involvement of autophagy in MBC-mediated HMGB1 inhibition, autophagy was pharmacologically inhibited by 3-methyladenine (Sigma-Aldrich, MO, Cat# M9281) as previously described [24].

### 2.7. Animal Model of Sepsis

 This study was approved and performed in accordance with the guidelines for the care and use of laboratory animals at the Feinstein Institute for Medical Research, Manhasset, NY. To evaluate the therapeutic potential of MBC extract, a clinically relevant animal model of sepsis induced by cecal ligation and puncture (CLP) was employed [14, 24, 32]. Briefly, the cecum of Balb/C mice was ligated at 5.0 mm from the cecal tip, and then punctured once with a 22-gauge needle [33]. MBC extract or saline was orally administered to mice at indicated doses and time points, and mice were monitored for survival for up to two weeks. 

### 2.8. HPLC and Mass Spectrometry Analysis

To gain insight into the molecular properties of active components responsible for inhibiting HMGB1 release, the aqueous MB coat extracts were fractionated by HPLC using a Nova-Pak C8 column (3.9 × 150 mm) and 0.065% trifluoroacetic acid (v/v, in water) as the mobile phase. The sample was eluted by a linear gradient of 0–59% acetonitrile (v/v, in 0.065% trifluoroacetic acid) over 22 min at a flow rate of 1.0 mL/min. The major components were monitored at UV wavelengths of 254, 280, 450, 360, and 550 nm. Each HPLC fraction was screened for HMGB1-inhibiting activities, and the components of the active HPLC fraction were analyzed by mass spectrometry using a triple quadrupole mass spectrometer (Thermo TSQ Quantum Access, Thermo-Fisher). 

### 2.9. Statistical Analysis

Data are expressed as mean ± SD of 2-3 independent experiments (*n* = 2-3). One-way ANOVA was used for comparison among all different groups. When the ANOVA was significant, post-hoc testing of differences between groups was performed using Tukey's test. The Kaplan-Meier method was used to compare the differences in mortality rates between groups. A *P* value <0.05 was considered statistically significant.

## 3. Results

### 3.1. MBC Extract Reduced Both Intra- and Extracellular HMGB1 Levels in Endotoxin-Stimulated Macrophages

**Figure 1:**
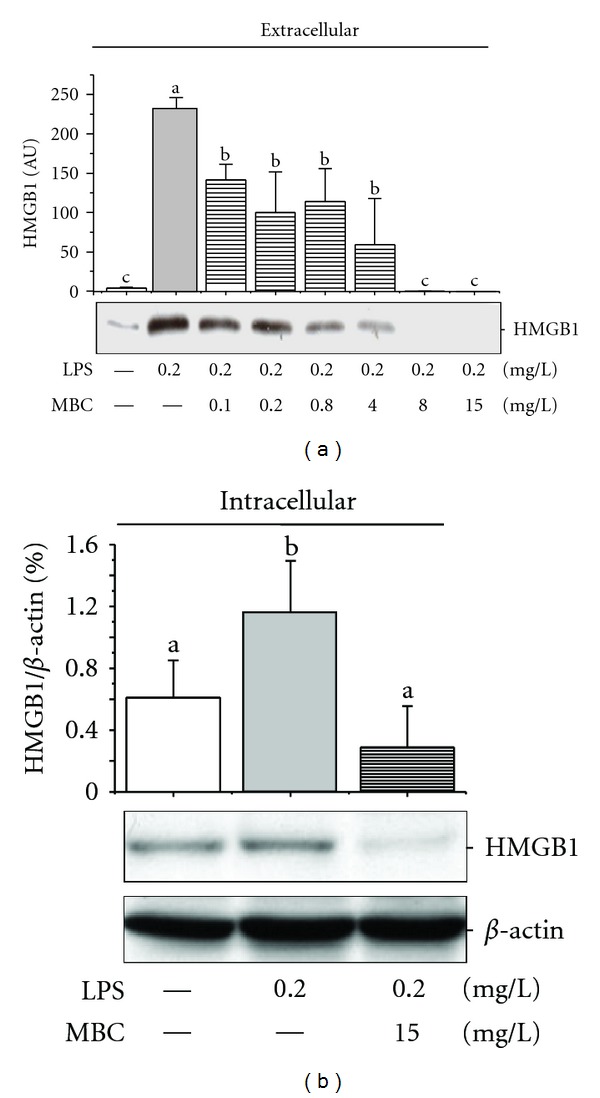
Mung bean coat (MBC) extract reduced bacterial endotoxin-induced HMGB1 release. Murine macrophages were stimulated with LPS in the absence or presence of MBC extract at indicated concentrations. At 16 h after LPS stimulation, levels of HMGB1 in the culture medium or whole cell lysate were determined by Western blotting analysis. Western blots shown were representative results of three independent experiments. Means not sharing a common letter differ, *P* < 0.05.

To verify whether mung bean possesses anti-inflammatory properties as historically described in traditional Chinese medicine, we first examined its effects on endotoxin-induced HMGB1 release in macrophage cultures. Indeed, the aqueous extract of whole mung bean significantly inhibited endotoxin-induced HMGB1 release (data not shown). To search for the active components, we prepared aqueous extracts of the MB coat and flesh separately, and examined each extract for anti-inflammatory activities. The aqueous extract of mung bean coat (MBC), but not the mung bean flesh (data not shown), dose-dependently reduced intra- and extra-cellular HMGB1 levels in endotoxin-stimulated murine macrophages (Figures 1(a) and 1(b)). It suggests that MBC extract not only effectively inhibited endotoxin-induced HMGB1 release, but also reduced cellular HMGB1 levels in primary macrophage cultures. 

### 3.2. Oral Administration of MBC Extract Rescued Mice from Lethal Sepsis

**Figure 2:**
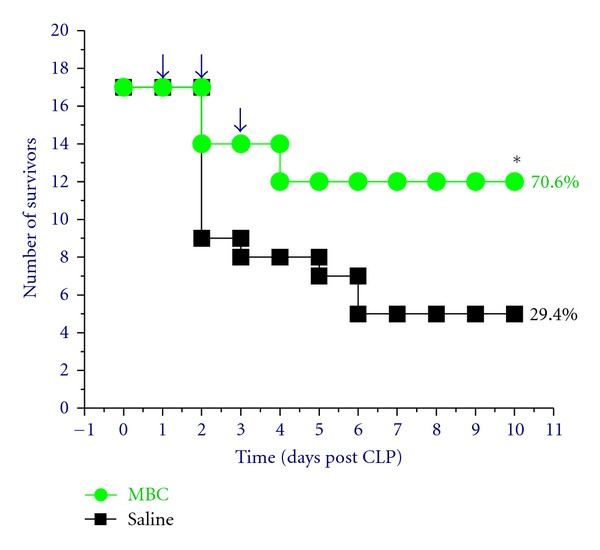
Oral administration of MBC extract significantly rescued mice from lethal sepsis. Balb/C mice were subjected to lethal sepsis (induced by cecal ligation and puncture, CLP). At +24, +48, and +72 hours post CLP, animals were orally administered with saline (0.2 mL/mouse) or MBC extract (0.2 mL/mouse, containing 1.0 mg lyophilized yellow substance), and animal survival rates were monitored for two weeks.

To appreciate the therapeutic benefits of MBC, we determined whether administration of MBC extract via a clinically feasible route confers protection in a clinically relevant model of sepsis. Oral administration of MBC extract (0.2 mL/mouse, containing 1.0 mg lyophilized MBC extract) at +24, +48, and +72 h post CLP, conferred a significant protection against lethal sepsis (Figure 2), increasing animal survival rates from ~30% to 70%. If given within 24 h of CLP, the timing of mung bean administration did not make any significant difference in improving animal survival rates. It supports a therapeutic potential for aqueous extract of MBC in the treatment of lethal experimental sepsis. 

### 3.3. MBC Extract Induced HMGB1 Aggregation and Autophagic Degradation

**Figure 3:**
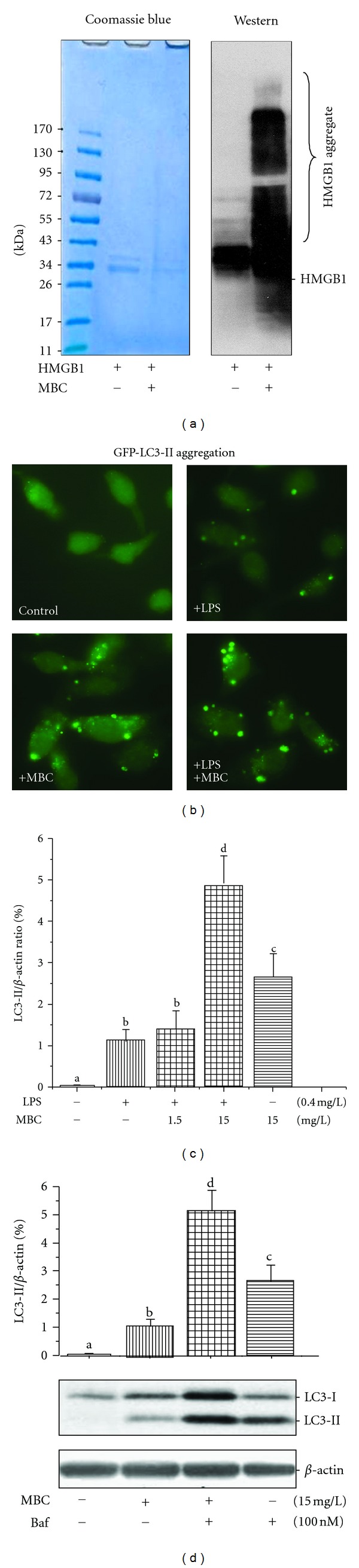
MBC extract induced HMGB1 aggregation and autophagy. (a) MBC extract induced HMGB1 aggregation *in vitro*. Highly purified HMGB1 protein (3 mg/L) was incubated with MBC extract (20 mg/L) for 1 h, resolved on SDS-PAGE gel, and stained by Coomassie blue or anti-HMGB1 antibodies (Western blotting). (b) MBC extract increased formation of LC3-containing vesicles. GFP-LC3-transfected RAW 264.7 cells were stimulated with LPS (0.2 mg/L) in the absence or presence of MBC extract (15 mg/L) for 16 h, and the formation of LC3 punctuates were examined under fluorescent microscopy. (c, d) MBC extract enhanced LC3-II production. Macrophages were stimulated with LPS or MBC extract in the absence or presence of an autophagy inhibitor, bafilomycin A1, for 16 h, and cellular LC3-II levels were determined by Western blotting. Means not sharing a common letter differ, *P* < 0.05.

To elucidate the mechanisms underlying MBC-mediated inhibition of HMGB1 release, we examined whether MBC extract induced HMGB1 aggregation. SDS-PAGE analysis revealed that incubation of HMGB1 with MBC extract led to the formation of SDS-resistant protein aggregates, which were recognized by HMGB1-specific antibodies as multiple bands with higher molecular weights (Figure 3(a)). Interestingly, the MBC-induced protein aggregation was completely prevented by coaddition of a reducing agent, 1,4-dithiothreitol (DTT, data not shown), suggesting that the MBC-induced HMGB1 aggregation was likely dependent on oxidative reactions. In addition, mung bean coat extract similarly induced aggregation of other proteins (e.g., recombinant human TNF, data not shown), indicating that MBC-induced protein aggregation is not entirely dependent on ionic interactions. 

 To test whether MBC extract, like other HMGB1 aggregation-inducing agents (e.g., EGCG) [24], affects autophagy, we determined the effect of MBC extract on the LC3-II aggregation and production in macrophage cultures. Consistent with previous reports [21, 24], LPS induced the formation of LC3-containing cytoplasmic vesicles (autophagosomes, Figure 3(b)), and elevated LC3-I to LC3-II conversion (Figure 3(c)). Even in the absence of LPS, MBC extract still induced the formation of LC3-containing punctate structures (Figure 3(b)), and elevated LC3-II levels in macrophage cultures (Figure 3(c)). To distinguish between the possibilities whether MBC extract elevated LC3-II production or merely decreased LC3-II degradation (i.e., autophagic flux), we determined the effect of bafilomycin A1, an inhibitor of autophagosome-lysosome fusion, on MBC-induced LC3-II elevation. Even in the presence of bafilomycin A1 at a saturating concentration for LC3-II accumulation (100 nM), MBC extract still significantly increased LC3-II levels (Figure 3(d)), suggesting that MBC extract increased autophagosome synthesis, rather than merely inhibiting LC3-II degradation (by reducing autophagosomes trafficking to, and fusion with, lysosomes) in human macrophages. 

**Figure 4:**
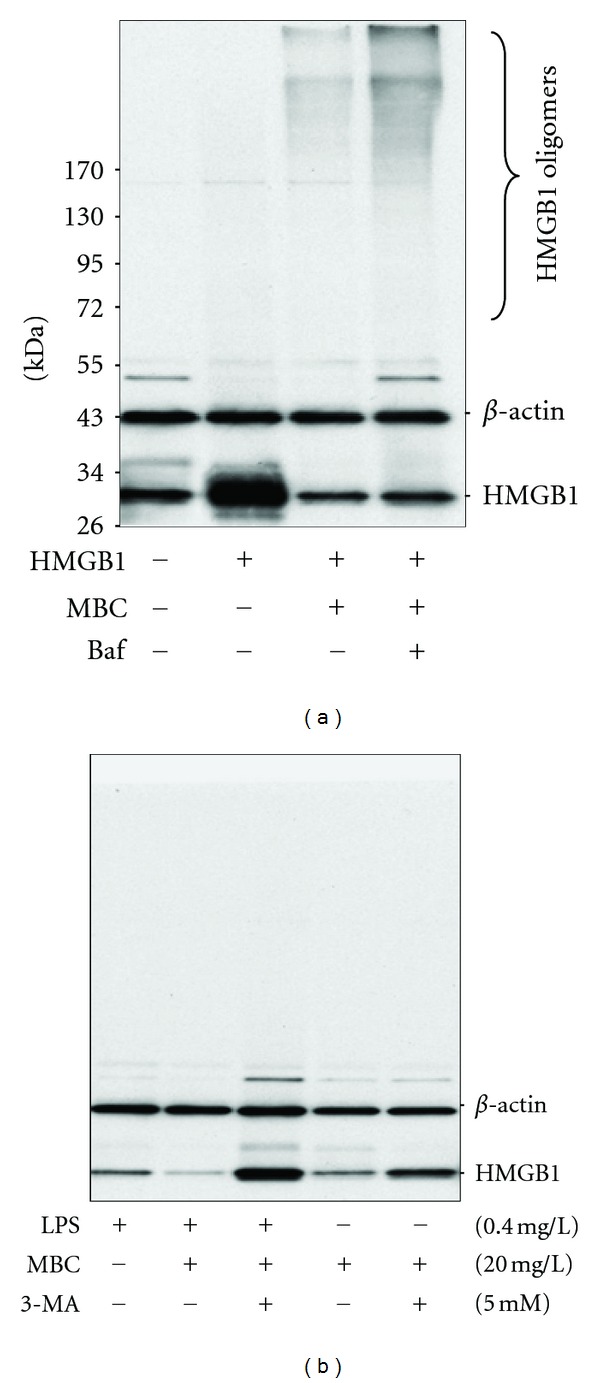
Autophagy inhibitors impaired MBC-mediated reduction of cellular HMGB1 levels. Differentiated human macrophages were incubated with highly purified HMGB1 protein (2 mg/L) or LPS (0.4 mg/L) in the absence or presence of MBC extract (20 mg/L), Baf (100 nM), or 3-methyladenine (5.0 mM), respectively. Cellular HMGB1 levels were determined by Western blotting analysis. The results shown were representative of three independent experiments.

 To test the possibility that MBC extract inhibits HMGB1 release by stimulating its autophagic degradation, we determined the effects of bafilomycin A1 on MBC-mediated reduction of intracellular HMGB1 levels. Consistent with a previous report [24], incubation of human macrophages with recombinant HMGB1 led to the elevation of cellular HMGB1 levels within 4–6 h (Figure 4(a)). However, the coaddition of MBC extract resulted in a decrease of HMGB1 levels, along with the appearance of HMGB1 aggregates in macrophage cultures (Figure 4(a)). At the concentration that effectively elevated cellular LC3-II levels (by inhibiting LC3-II degradation), bafilomycin A1 also markedly elevated levels of HMGB1 mono- and oligomers (Figure 4(a)). It suggests that MBC extract may inhibit HMGB1 release by stimulating autophagic HMGB1 degradation. To confirm this possibility, we also examined the effect of another autophagy inhibitor for autophagy-regulating signaling molecule (PI3K-class III), 3-methyladenine (3-MA), on cellular HMGB1 levels in endotoxin-stimulated macrophage cultures. Similarly, the MBC-mediated decrease in cellular HMGB1 levels was dramatically impaired by 3-MA (Figure 4(b)), confirming that MBC extract inhibits HMGB1 release by stimulating its autophagic degradation.

### 3.4. Characterization of Active Components of MBC Extract

**Figure 5:**
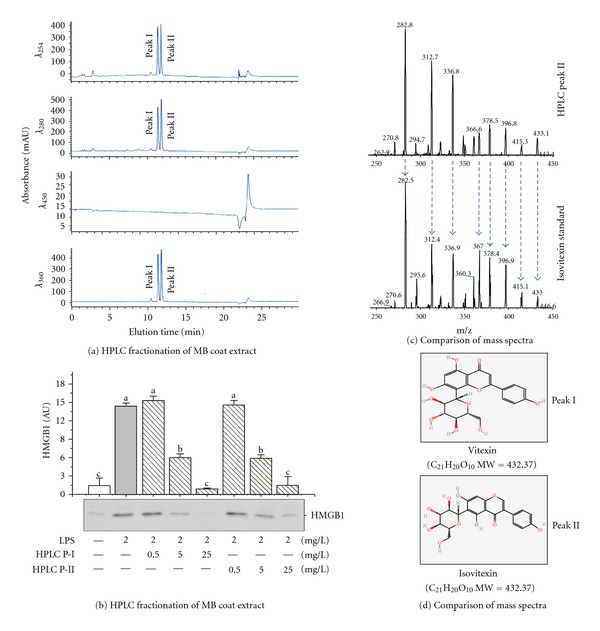
Characterization of active components of MBC extract. (a, b) HPLC fractionation of MBC extract. MBC extract was fractioned by HPLC, and each fraction was screened for activity in inhibiting endotoxin-induced HMGB1 release. Note that two major UV absorption peaks (I and II) were detected at the wavelengths of 254, 280, and 360 nm (a) and coeluted with the activities to inhibit endotoxin-induced HMGB1 release (b). (c, d) Mass spectrometry analysis of HPLC peaks. The MS spectrum of the MBC HPLC peak II was identical to that of the commercially obtained isovitexin standard.

To gain insights into the chemical properties of the active components, we fractionated the MBC extract by HPLC, and screened each fraction for HMGB1-inhibiting activities. The major HMGB1-inhibiting activities were coeluted with two major HPLC peaks detected at wavelengths of 254, 280, and 360 nm (Figures 5(a), 5(b)). Mass spectrometry analysis revealed the presence of two substances with an identical *m*/*z* = 433.09 (100.0%), corresponding to an empirical formula of C_21_H_20_O_10_. These chemical properties agreed with those of previously identified mung bean major flavonoids isomerides: vitexin and isovitexin [34]. Consistently, the mass spectra of the major HPLC fractions were identical to those of the commercially obtained vitexin (data not shown) and isovitexin standards (Figure 5(c)). Taken together, these data suggested that the major active MBC components responsible for inhibiting HMGB1 release were coeluted with the two major MB flavonoids: vitexin and isovitexin. 

### 3.5. Anti-Inflammatory Properties of MBC Flavonoids

**Figure 6:**
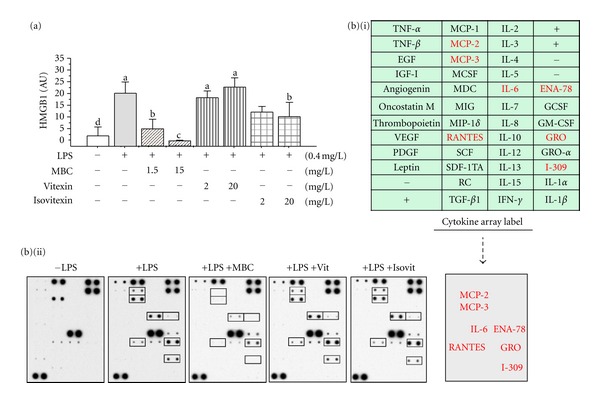
MBC extract inhibited the release of HMGB1 and multiple chemokines in human macrophages. Differentiated human macrophages were stimulated with LPS in the absence or presence of MBC extract, vitexin, or isovitexin at indicated regimens and assayed for extracellular levels of HMGB1 and other cytokines by Western blotting analysis (a) or Cytokine Antibody Arrays (b), respectively. (b) (i) denotes the names of cytokines on the Cytokine Antibody Arrays. Means not sharing a common letter differ, *P* < 0.05.

We then examined the effects of MBC flavonoids on the release of HMGB1 and 42 cytokines/chemokines in endotoxin-stimulated human macrophages. As shown in Figure 6, MBC extract similarly inhibited endotoxin-induced HMGB1 release in a dose-dependent manner (Figure 6(a)). Furthermore, MBC extract also dramatically inhibited endotoxin-induced secretion of IL-6, as well as several chemokines (MCP-2, MCP-3, RANTES, GRO, and I-309) (Figure 6(b)). Surprisingly, only isovitexin moderately suppressed endotoxin-induced HMGB1 release (Figure 6(a)), and did not affect endotoxin-induced release of other cytokines/chemokines (Figure 6(b)). 

## 4. Discussion

Many traditional medicinal herbs have been successfully developed into effective therapies for various inflammatory ailments. For instance, the use of willow bark extract to reduce pain and fever was documented by a Greek physician (Hippocrates) in the 5th century BC. The subsequent discovery of salicylic acid as its pain/fever-relief active component gave rise to the first synthetic nonsteroidal anti-inflammatory drug (NSAID) aspirin, and the birth of the pharmaceutical industry. Similarly, a Chinese herb, *Artemisia annua*, has been documented in the treatment of malaria in the 4th century. The recent identification of artemisinin as its antimalarial active component led to the development of a standardized effective therapy for malaria worldwide [35]. Recently, we and others demonstrated that the major components of several herbs, including Danggui [28], Danshen [13], and green tea [23, 24], red grape [36, 37], and marijuana [38–40], conferred protection against infection- or injury-elicited inflammatory responses. In the present study, we discovered that the aqueous extract of MBC effectively inhibited endotoxin-induced HMGB1 release, and rescued mice from lethal sepsis even when given orally at 24 h post the onset of sepsis. The doses of MBC extract given to septic mice (40 mg/kg) are comparable to those achievable in humans (with an average body weight of 75 kg) after ingestion of 285 g mung bean. Fortunately, the major active components were predominantly enriched in the MB coats, so an individual does not have to consume large amount of mung bean to enjoy its medicinal benefit. In fact, the active components could be easily extracted from MBC in hot water, making it feasible for clinical use in the treatment of sepsis and other inflammatory diseases. 

 The underlying mechanisms by which MBC extract conferred protection against lethal sepsis are complex, but may be attributable to the inhibition of endotoxin-induced release of HMGB1, IL-6, and several chemokines (including MCP-2, MCP-3, RANTES, GRO, and I-309). Although appropriate production of various cytokines/chemokines is essential for the immunity against infection (by allowing leukocyte activation and recruitment to sites of infection), exaggerated production of these cytokines or chemokines may contribute to the pathogenesis of sepsis and other inflammatory diseases. In addition, co-addition of MBC led to a >50% reduction of bacterial colony formation units, implicating a possibility that MBC might possess direct bactericidal activities. It will thus be important to investigate whether MBC confers protection against sepsis by attenuating systemic inflammation or affecting bacterial elimination in future studies.

Furthermore, we provided evidence for a novel mechanism by which MBC extract inhibits endotoxin-induced HMGB1 release: it induces HMGB1 aggregation and subsequent autophagic degradation. At the concentrations effective for inhibiting LPS-induced HMGB1 release, MBC extract stimulated LC3 punctate (autophagosome) formation and LC3-II elevation, even in the presence of bafilomycin A1 at concentrations that completely inhibited autophagosome-lysosome fusion. Thus, MBC extract was capable of stimulating autophagosome synthesis, rather than simply inhibiting LC3-II degradation. The mechanism by which MBC extract induces autophagy may relate to its capacity in inducing the formation of SDS-resistant HMGB1 aggregates. Because large HMGB1 complexes can not physically pass through the narrow pore of the proteasome barrel of the ubiquitin-proteasome pathway, they may consequently trigger another cellular degradation process, autophagy [24]. Notably, it was recently suggested that cytoplasmic HMGB1 may interact with beclin-1 and function as an important regulator of autophagy [41, 42]. It will thus be important to investigate whether HMGB1 is essential in MBC-mediated autophagy in innate immune cells.

 As a potential consequence, MBC extract may induce HMGB1 degradation via an autophagosome-lysosome-dependent pathway. This possibility was supported by the observation that MBC extract simultaneously prevented LPS-induced HMGB1 release, as well as elevation of intracellular HMGB1 levels in primary macrophage cultures. Furthermore, cotreatment with autophagy inhibitors (such as 3-MA and bafilomycin A1) dramatically impaired MBC-mediated reduction of cellular HMGB1 levels.

 The identification of the active components of medicinal herbs has always been a barrier for herbal remedies to enter the pharmaceutical industry. This is particularly true when the effectiveness of an herbal remedy rely on the complex interactions between many constituents. The active components responsible for MBC-mediated HMGB1 release remain to be elucidated. The major HMGB1-inhibiting activities were coeluted with two major HPLC peaks that contained substances sharing identical mass spectrum with vitexin and isovitexin. Paradoxically, our HPLC-purified MBC components were easily solubilized in water, whereas commercially obtained vitexin or isovitexin was barely water-soluble and needed to be dissolved in organic solvent (e.g., DMSO). Even though the commercially obtained flavonoids could slightly induce HMGB1 protein aggregation and moderately inhibited endotoxin-induced HMGB1 release, their relative capacities were dramatically lower than those of the MBC crude extract or purified HPLC fractions. The molecular basis for these dramatic activity differences between our HPLC-purified and commercially obtained flavonoids remains an intriguing subject of future investigations.

## 5. Conclusions

Here we have validated the therapeutic potential of MBC extract in a clinically relevant animal model of sepsis by administering it orally in a delayed regimen. Furthermore, we have uncovered a novel mechanism by which MBC extract effectively inhibits HMGB1 release by inducing HMGB1 aggregation and autophagic degradation in macrophages. Given the shared role of HMGB1 in the pathogenesis of many diseases, it is tempting to consider that MBC consumption may be beneficial against pathogenic heat, oxidative stress, or lethal infections in humans by selectively modulating autophagic HMGB1 degradation in targeted cells. Further investigation in this area will improve our understanding of innate immune-modulating mechanisms of mung bean, and shed light on the development of novel autophagy-modulating therapeutic strategies for the treatment of human diseases. 

## Abbreviations

CLP: Cecal ligation and puncture
HMGB1: High mobility group box 1 protein
LPS: Lipopolysaccharide
MBC: Mung bean coat
LC3: Microtubule-associated protein 1 light chain 3.
